# Blood purine measurements as a rapid real-time indicator of reversible brain ischaemia

**DOI:** 10.1007/s11302-017-9578-z

**Published:** 2017-08-12

**Authors:** Faming Tian, Fakhra Bibi, Nicholas Dale, Christopher H. E. Imray

**Affiliations:** 1 Sarissa Biomedical Ltd., Vanguard Centre Sir William Lyons Road, Coventry, CV4 7EZ UK; 2 School of Life Sciences, University of Warwick, Coventry, CV4 7AL UK; 3 Department of Vascular Surgery, University Hospitals of Coventry and Warwickshire, Clifford Bridge Road, Coventry, UK; 4 Warwick Medical School, Coventry, CV4 7AL UK

**Keywords:** Adenosine, Brain Ischaemia, Carotid artery, Cerebral blood flow, Neurosurgery

## Abstract

To preserve the disequilibrium between ATP and ADP necessary to drive cellular metabolism, enzymatic pathways rapidly convert ADP to adenosine and the downstream purines inosine and hypoxanthine. During ischaemia, these same pathways result in the production of purines. We performed a prospective observational study to test whether purine levels in arterial blood might correlate with brain ischaemia. We made real-time perioperative measurements, via microelectrode biosensors, of the purine levels in untreated arterial blood from 18 patients undergoing regional anaesthetic carotid endarterectomy. Pre-operatively, the median purine level was 2.4 μM (95% CI 1.3–4.0 μM); during the cross-clamp phase, the purines rose to 6.7 μM (95% CI 4.7–11.5 μM) and fell back to 1.9 μM (95% CI 1.4–2.7 μM) in recovery. Three patients became unconscious during carotid clamping, necessitating insertion of a temporary carotid shunt to restore cerebral blood flow. In these, the pre-operative median purine level was 5.4 μM (range 4.7–6.1 μM), on clamping, 9.6 μM (range 9.4–16.1 μM); during shunting, purines fell to below the pre-operative level (1.4 μM, range 0.4–2.9 μM) and in recovery 1.8 μM (range 1.8–2.6 μM). Our results suggest that blood purines may be a sensitive real-time and rapidly produced indicator of brain ischaemia, even when there is no accompanying neurological obtundation.

## Introduction

Cellular metabolism is driven by the disequilibrium between ATP and ADP [1]. While glycolysis and oxidative phosphorylation ultimately generate ATP, enzymatic pathways exist in cells to rapidly remove ADP and, in effect, convert it to adenosine and the downstream purines inosine and hypoxanthine. For every molecule of ATP used by cellular processes, a molecule of adenosine is rapidly generated. Indeed, this can be observed in real time in brain tissue in vitro [2]. This rapid conversion of ADP to downstream metabolites helps to maintain the disequilibrium between ATP and ADP.

When cells are provided with insufficient substrates (e.g. glucose or lactate) and/or O_2_ to make ATP, the enzymatic systems that maximize the disequilibrium between ATP and ADP will rapidly cause the intracellular accumulation of adenosine and downstream purines. These metabolites can then efflux via equilibrative transporters [3] into the extracellular space. This ischemic production of extracellular adenosine and purines has been observed in brain tissue both in vivo and in vitro [4–14].

The blood-brain barrier (BBB) preserves the extracellular environment of the brain to provide stable conditions for neural computation [15–17]. The BBB also regulates the efflux of chemicals from the brain interstitial space to the blood via the presence of specialized transporters [18]. While purine nucleosides can enter the brain from blood [19], it is by no means certain that the efflux of purines into the extracellular space could be sufficient to result in an increase in their concentration in blood. Although a few studies have examined whether purine levels in blood increase during stroke [20] and brain ischaemia [21], this has not been widely studied. To address this issue, we have used a novel biosensor technology to examine the purine levels in sequential arterial blood samples taken before, during and after the mild and reversible brain ischaemia that is imposed during the cross-clamp phase of awake carotid endarterectomy. This timed, controlled, ischaemic insult to the brain provides an ideal model in which to investigate how rapidly purines are released into the blood following the onset of ischaemia, and how quickly they return to baseline following resolution of the ischemic insult.

## Materials and methods

### Patient selection

Patients undergoing elective awake carotid endarterectomy (CEA) were studied between May 2012 and November 2014. Patients were referred from the local TIA clinic, and were assessed within a vascular surgical clinic. The surgery was performed by a single surgeon (CHEI) at the University Hospital Coventry and Warwickshire.

### Surgical procedures

All CEAs were performed under loco-regional anaesthesia. The anaesthetic was administered by one of two experienced vascular anaesthetists with the aim being to personalize each patient’s care, keeping them pain free and physiologically stable while using the minimum necessary sedation. Local anaesthetic drugs included bupivacaine and lignocaine, and the short acting intravenous opioid remifentanil was used intermittently, as was the sedative midazolam. A combination of intravenous glycerol trinitrate and metaraminol were used to maintain the systolic blood pressure between 110 and 180 mmHg. The surgical procedures were carried out with 3.5 times optical magnification, and a selective shunt and patch policy was adopted. Prior to clamping of the carotid arteries, intravenous heparin was administered, using a fixed dose of 4000 units. Post-operatively, the patients recovered overnight in a postanaesthetic care unit (PACU). Transcranial Doppler was used to assess post-operative cerebral microembolisation [22].

### Neurological assessment

Shunting was determined by awake-testing, the indication for shunting being profound neurological obtundation, significant confusion, restlessness or inability to respond to commands as determined by continuous clinical assessment by the anaesthetist. Profound deterioration that occurred within the first 90 s was handled by declamping the common carotid artery and allowing the deficit to recover. The operation was then continued under general anaesthesia, and the carotid shunt was inserted in a controlled fashion. A deficit that occurred more than 90 s after cross-clamp, but before the carotid arteriotomy (trial clamp for 5 min), was handled by temporary clamp release. Once normal neurology was restored, clamps were then reapplied allowing a shunt to be inserted before the patient became obtunded a second time [23].

### Blood sampling

An arterial line was inserted under local anaesthetic (lignocaine 1%) into the contralateral radial artery for routine intraoperative and post-operative blood pressure monitoring. Blood samples were drawn from this line in the anaesthetic room prior to surgery, during the exposure phase, prior to cross-clamping, during the cross-clamp phase, post clamp release, during closure and in the PACU.

### Biosensor measurements

Measurements were performed in a prep room adjacent to the operating theatre, giving real-time analysis at the point of care. We used microelectrode biosensors previously described [24] to measure the purines in fresh unprocessed blood. In brief, these gold electrodes are coated with a Ruthenium Purple layer which acts as a mediator to provide the necessary selectivity against interferences such as ascorbate, urate and acetaminophen [24]. This allowed the accurate measurement of purines in whole blood.

**Fig. 1:**
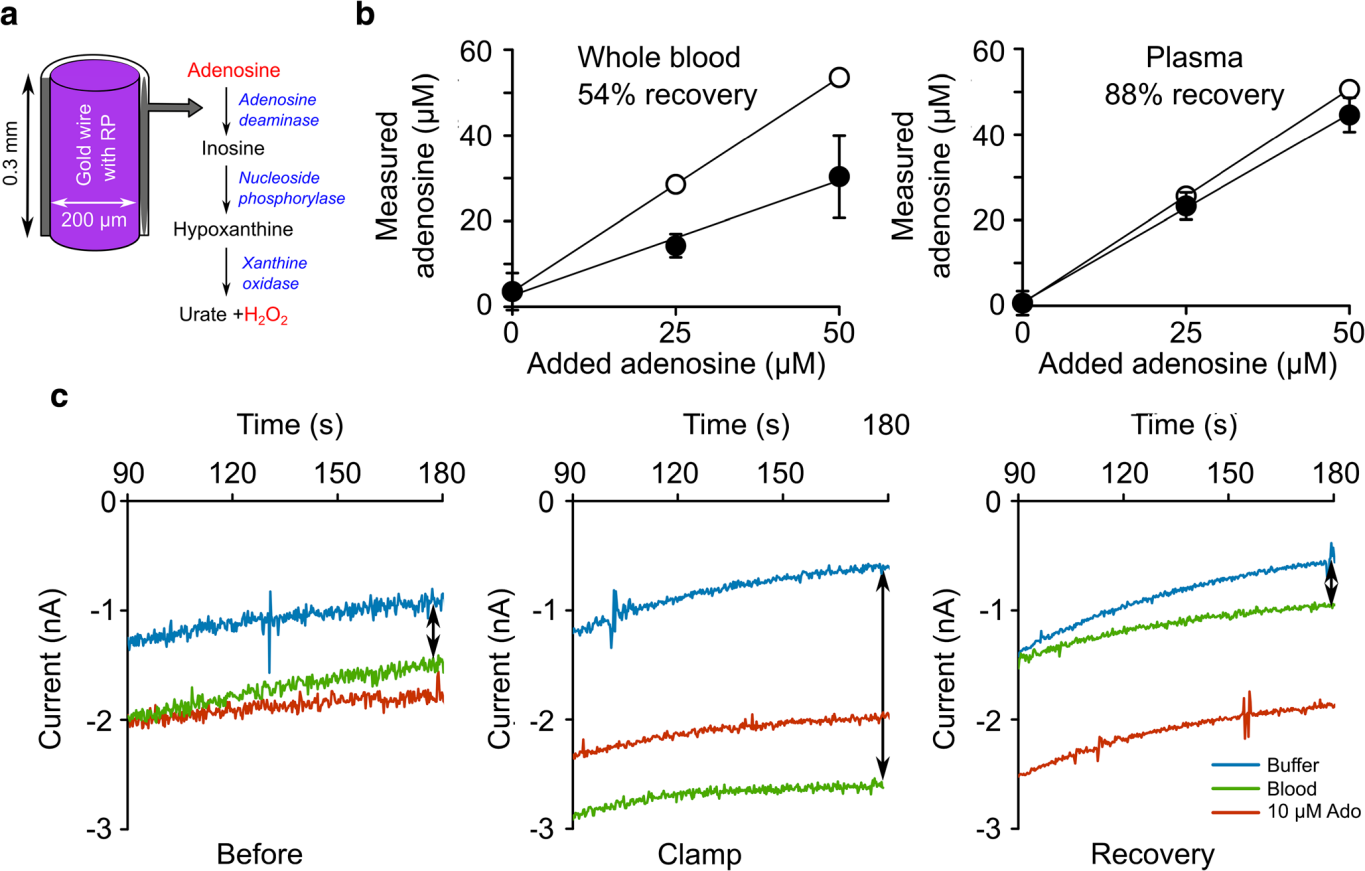
Measurement of purines in arterial blood. **a** Enzymatic cascade used to detect the blood purines. The enzymes are entrapped within a layer on a Ruthenium Purple (RP)-coated gold electrode. **b** Demonstration of analytical performance of biosensor in whole blood (*left*) and plasma (*right*). A measurement of baseline purines was made and then known concentration of adenosine added to the sample. The *open circles* show the expected values of the measurements (baseline purines plus the known added amount). The *filled circles* show the amount of adenosine measured with the biosensor. **c** Example records of sensor currents during blood measurements from a type 3 patient showing the pre-operative blood purine level, shortly after carotid clamping, and following recovery. The current records show the last 90 s of the measurement and are the difference between the purine and null biosensors. The charging current in buffer (*blue*) indicates the “zero current” level, the *red trace* shows the calibration with 10 μM adenosine and the *green trace* is the measurement of the blood. The purine concentration in blood is calculated by taking the difference between the blood and buffer traces (*black double-headed arrows*), and expressing this as a proportion of the difference between the calibration and buffer traces

The purine sensor has an enzymatic layer containing a cascade of three enzymes (Fig. 1a), which allowed it to detect all of the substrates for these enzymes: adenosine, inosine, hypoxanthine and xanthine [24, 25]. We made amperometric measurements to detect the electroreduction of peroxide produced by the final enzyme in the detection cascade, xanthine oxidase. We also made “null” biosensor recordings as a control comparison. The null biosensors were identical to the purine biosensors in all respects except that they lacked the enzymatic cascade and therefore could not respond to the purines [12].

We verified the accuracy of these sensors by measuring known amounts of added purines in whole blood and plasma (Fig. 1b). In whole blood, where red blood cells can take up the purines and have abundant degradative enzymes on their plasma membranes, the half-life of adenosine is reported to be in the order of seconds while that of the downstream purines, inosine and hypoxanthine, can be in the order of minutes [26–28]. In whole blood, as might be expected, we only recovered about 54% of the added adenosine (Fig. 1b). However, in plasma, where the blood cells have been spun down, the purines are much more stable and we measured about 88% of the added purines (Fig. 1b). As the electrochemical interferences in whole blood and plasma are the same, these results confirm that the biosensors are highly selective [24]. They also emphasize the need for rapid point-of-care purine measurements to exploit purines as indicators of clinical pathology as they are very unstable in whole blood. Our ultimate aim in developing these biosensors was to facilitate the use of purine measurements to inform clinical decision-making processes. Whole blood is routinely used clinically for a range of diagnostic tests at the point of care. Therefore, despite the likely partial loss of purines from whole blood, we performed this study on fresh unprocessed blood to gain proof of principle of clinical utility.

To measure purines in whole blood from the patients, the null and purine biosensors were introduced into the blood sample as soon as possible after sampling. They were simultaneously polarized to the working potential of −50 mV (versus Ag/AgCl), and the amperometric faradaic charging currents recorded (Fig. 1c). After 3 min, the current value of the null sensor was subtracted from the purine biosensor to give the “purine current”. This was converted into a purine concentration by comparing it to the current obtained from calibrating the sensors in a known amount of adenosine (Fig. 1c).

### Statistical presentation and analysis

All data are presented as medians with 95% confidence limits—in the case of the smaller subgroups of the data (type 1, 2 and 3 patients), the 95% confidence limits are the same as the range of the data. For the entire group, the data was analysed in two ways: (1) in a two-way Friedman ANOVA comparing the preclamp, clamp and recovery purines across the phases of the procedure within each patient; and (2) as cumulative probability distributions of the purine levels at each phase of the procedure—the medians and distributions being compared via the Mann-Whitney *U* test and the Kolmogorov-Smirnov tests respectively.

## Results

**Fig. 2:**
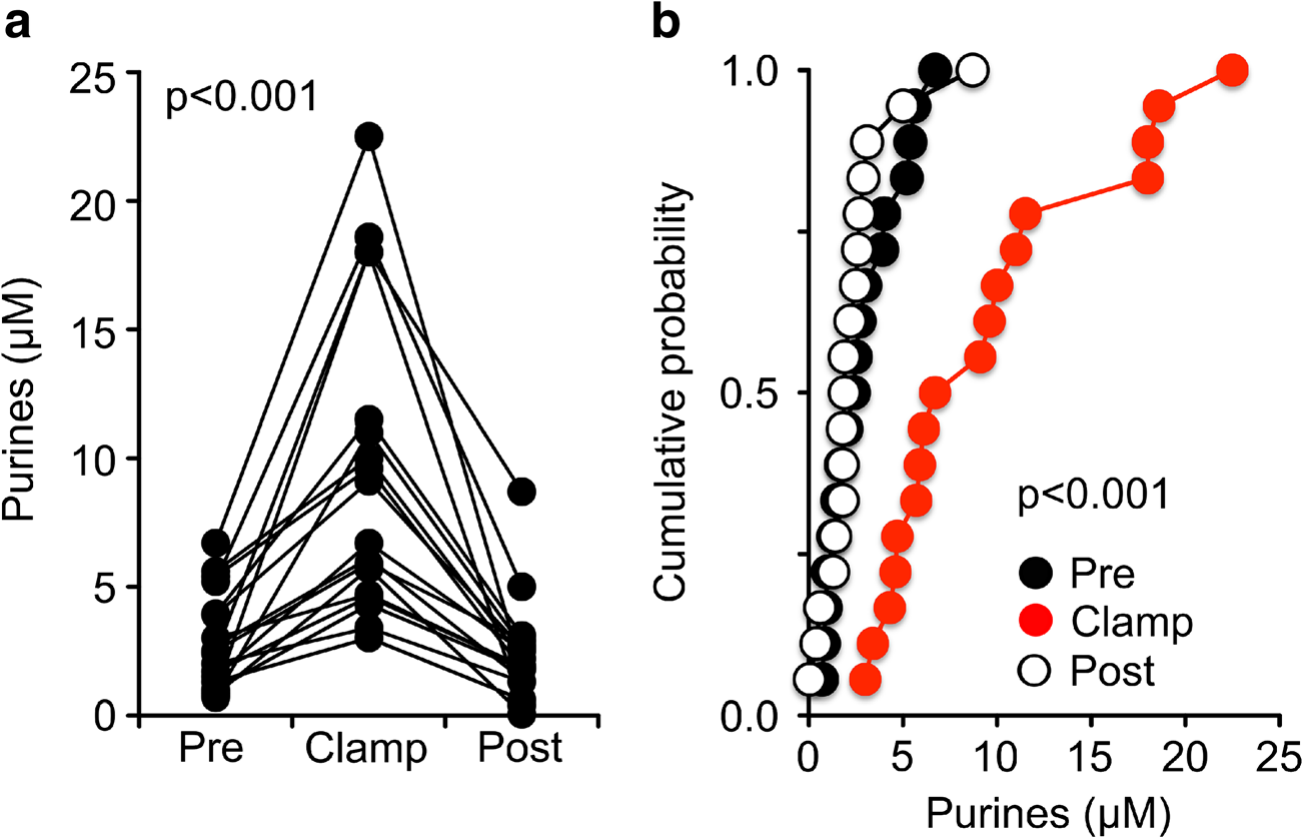
Purines in arterial blood increase during carotid clamping. **a** Plot of purine levels in the pre-operative (Pre), carotid clamp (Clamp) and after recovery (Post) for each patient. In every patient, blood purine levels increase relative to the pre-operative baseline. Statistical comparison performed with the Friedman two-way ANOVA. **b** The same data plotted as cumulative probability distributions for the pre-operative, carotid clamp and recovery phases. Note that purine levels return to pre-operative levels in the recovery phase. Statistical comparison of medians performed with the Mann-Whitney *U* test. A Kolmogorov-Smirnov comparison of the cumulative probabilities of the pre-operative and clamp purine levels gives *D* = 0.6667, with *p* = 0.000

Over a period of 2 years, we collected measurements from 18 patients. We first analysed the data obtained from these patients as a single group. Overall, the median resting purine level in blood, measured pre-operatively, was 2.4 μM (1.3 to 4.0 μM, Fig. 2). This value is comparable to others in the literature which suggest that plasma concentrations of hypoxanthine/xanthine in humans (the predominant purines in blood) are in the range of 1–2 μM [29, 30]. During carotid clamping, the blood purine levels rose in every patient relative to the pre-operative baseline (Fig. 2a). This increase was equally apparent when the data for the blood levels in the pre-operative, clamp and recovery periods were plotted as cumulative probability distributions (i.e. treating the three conditions as independent samples, Fig. 2b). The median purine level in the clamp phase was 6.7 μM (4.7 to 11.5 μM). This represents a 2.8-fold increase in concentration during the clamp phase compared to the baseline. Following recovery, the blood purines fell to the pre-operative baseline (1.9 μM, 1.4 to 2.7 μM). Our analysis shows that within a relatively short period following release of the clamp (less than 1.5 h), the blood purine levels were indistinguishable from the pre-operative baseline.

We made repeated sequential measurements of blood purines throughout the carotid operative procedure. Inspection of the profile of these measurements, combined with the concomitant neurological assessment of the patients, led us to identify three patterns of perioperative purine release: type 1, type 2 and type 3. Patients that displayed the type 1 and 2 patterns (7/18 and 8/18 respectively) displayed no major neurological symptoms during carotid clamping. However, patients displaying the type 3 pattern of purine release (3/18) rapidly became unconscious following the clamping of the carotid artery.

**Fig. 3:**
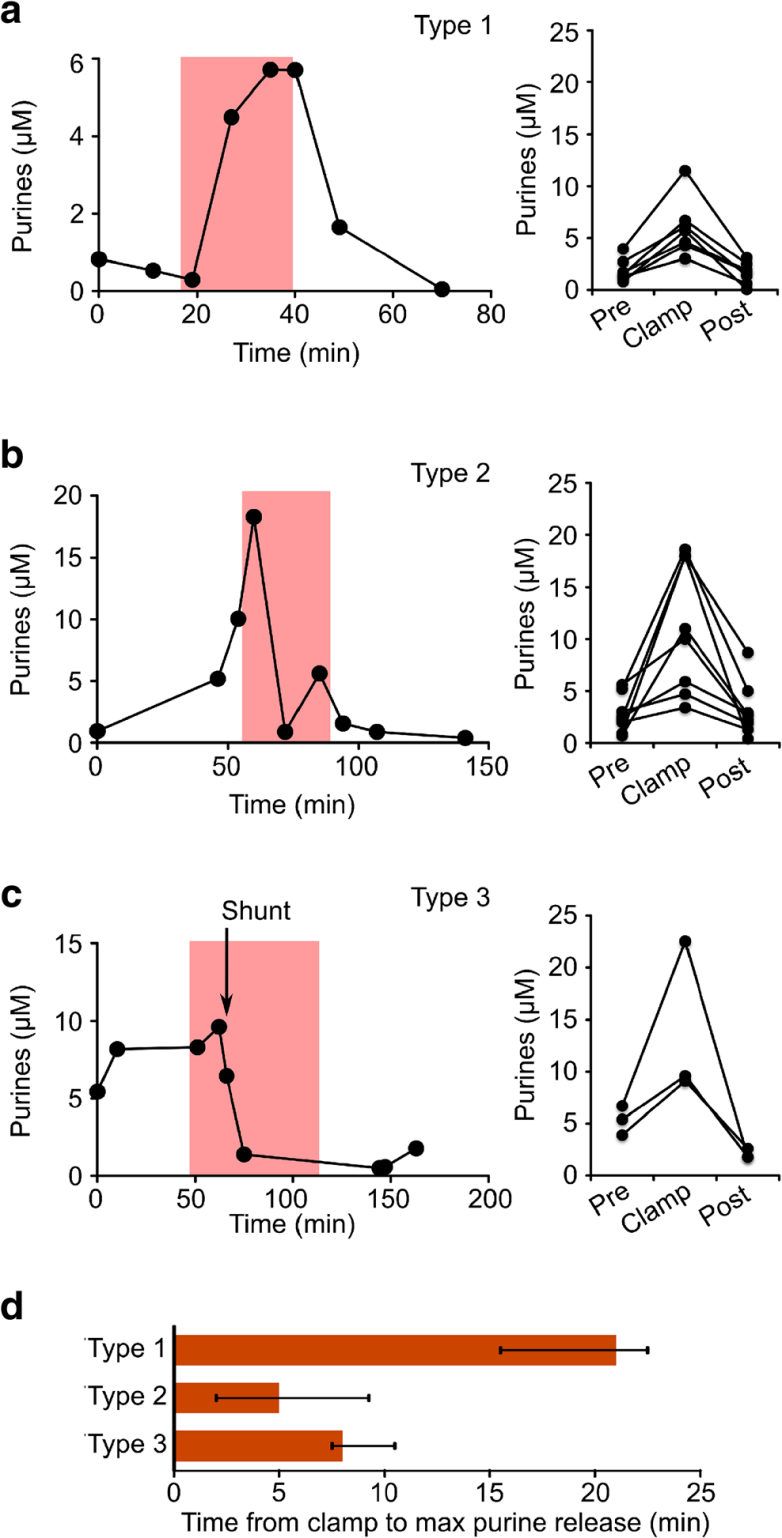
Sequential blood purine measurements during carotid endarterectomy reveal different profiles of purine release. **a** *Left*, example of sequential measurements of blood purine levels during a type 1 pattern. The *pink rectangle* indicates the timing and duration of the carotid clamping. *Right*, plots of the pre-operative, carotid clamp and recovery levels of purines for all patients displaying the type 1 pattern. **b** *Left*, example of sequential measurements of blood purine levels during a type 2 pattern. *Right*, plots of the pre-operative, carotid clamp and recovery levels of purines for all patients displaying the type 2 pattern. **c** *Left*, example of sequential measurements of blood purine levels during a type 3 pattern. Note how purine levels rapidly dropped following installation of the carotid shunt (*arrow*). *Right*, plots of the pre-operative, carotid clamp and recovery levels of purines for all patients displaying the type 3. **d** Histograms of the median time from carotid clamp to maximal recorded purine release during the clamp phase for patients displaying the type 1, 2 and 3 patterns. *Error bars* are upper and lower quartiles

During the type 1 pattern, the rise in purine levels was sustained throughout the clamp period and reached its maximum towards the end of the clamp period (Fig. 3a). In these patients, the median time to maximal purine blood level was 21 min (14 to 29 min, Fig. 3d). The median pre-operative purine level was 1.5 μM (0.8 to 4.0 μM) in patients displaying the type 1 pattern. During carotid clamping, purines rose to 5.7 μM (3 to 11.5 μM). This represents a 3.8-fold increase in purine concentration during the clamp phase compared to the baseline. During the recovery period, purines in blood fell to 1.8 μM (0.05 to 3.1 μM, Fig. 3a).

Patients displaying the type 2 pattern had pre-operative baseline purines of 2.4 μM (0.7 to 5.6 μM), and they exhibited a transient pattern of purine release during the carotid clamping. Their blood purines reached a peak (10.0 μM, range 3.4 to 18.6 μM) much quicker than during the type 1 pattern (3 min, 1 to 16 min, *p* = 0.001, Mann-Whitney *U* test compared to type 1 pattern, Fig. 3b, d). This was a 4.2-fold increase in purine concentration during the clamp phase compared to the baseline. After reaching this peak, the blood purine levels declined, but in six of eight cases remained higher than the pre-operative baseline (3.2 μM, 0.9 to 13.6 μM). On recovery, the purine levels returned to 2.2 μM (0.4 to 8.7 μM, Fig. 3b).

During the type 3 pattern, patients rapidly became unconscious following carotid clamping. Although we only observed three patients displaying this type of pattern, they were extremely interesting. The baseline purine levels were elevated compared to those seen during the type 1 and type 2 patterns (5.4 μM, 3.9 to 6.7 μM, Figs. 1b and 3c). The purines rose on clamping to 9.6 μM (9.1 to 22.5 μM). The increase in purines following the clamp phase was 1.8-fold compared to the baseline. During recovery following the procedure, they fell to 1.8 μM (1.8 to 2.6 μM). This recovery value was lower than the pre-operative baseline value. Importantly, when the carotid artery was shunted to restore cerebral blood flow, the blood purine levels dropped to below the pre-operative baseline levels (1.4 μM, 0.4 to 2.9 μM, Fig. 3c). This observation suggests that the brains of these patients were chronically ischaemic owing to impeded carotid blood flow, which was relieved initially by the temporary shunt (hence the purine levels fell), and also in the longer term by the outcome of the operation.

## Discussion

CEA is performed to reduce the risk of a future stroke. An integral part of the procedure is a temporary cross-clamping of the carotid artery. As the timing and release of the carotid clamp and hence the ischaemic insult are defined, this procedure provides an excellent opportunity to test in human patients whether purine levels in blood are a marker of cerebral ischaemia. In all 18 patients, we found that purine levels in arterial blood rose within minutes of applying the carotid clamp. In most patients, this occurred in the absence of any overt neurological indication. This would suggest that the purines are a very sensitive measure of cerebral ischaemia. Elevated purine levels were observed throughout the clamp period, demonstrating that the purines are continually produced and released from the brain while the ischaemic insult persists. Following release of the clamp, the blood purine levels returned relatively quickly (within 1–2 h) to the pre-clamp baseline. The purines are thus a relatively short-lasting indication of cerebral ischaemia.

The ischaemic insult resulting from the carotid cross-clamping is relatively mild and diffuse. Unlike a stroke, this insult will not be expected to be accompanied by either cell death or damage to the blood-brain barrier. Nevertheless, in every case during carotid cross-clamping, we detected elevated purines in arterial blood. This observation has two important implications: (1) metabolically stressed, but otherwise healthy, cells in the brain release purines into the extracellular fluid; and (2) under physiological conditions, the blood-brain barrier can transport sufficient purines to enable their detection in blood. We suggest therefore that the purines could be used, firstly, to detect the incidence of cerebral ischaemia from its earliest origins and, secondly, to monitor the persistence of the ischaemic insult and finally to assess resolution. As we have used biosensors to measure the purines in freshly drawn untreated blood, our methods may comprise the basis of a point-of-care blood test for the diagnosis and real-time monitoring of stroke.

The release of adenosine during CEA has been previously studied [21]. There are important methodological differences between the prior study and our study. Firstly, in the previous study the patients underwent the procedure under general anaesthetic, and neurological well-being was assessed indirectly by a somatosensory evoked potential in the cerebral cortex. Secondly, blood samples were taken from the jugular venous outflow. Thirdly, adenosine levels were measured by HPLC methods, which necessitated the storage and processing of the blood sample. The previous study only observed adenosine release if there was a loss of the somatosensory evoked potential following carotid cross-clamping.

In our study, we have used a more sensitive measurement method that uses peripheral blood sampling, and does not require the processing or storage of blood thus giving real-time measurements. We observed increased purine release in every patient undergoing the procedure, and were able to construct a profile of the release pattern via sequential measurements. Nevertheless, our study supports the conclusion of the previous study that purines are a sensitive marker of cerebral ischaemia.

We separated the perioperative purine profiles observed in patients into three groups based on the dynamics of purine release and whether the patients lost consciousness. Although this separation is purely empirical, we tentatively propose an underlying mechanistic basis. Patients displaying the type 1 pattern exhibited a rather slower increase in blood purines that was relatively modest in magnitude. They may therefore have retained a higher ability to compensate for the loss of blood flow from the ipsilateral carotid artery by enhancing flow from the contralateral side via the Circle of Willis than the patients displaying the other patterns of purine release. During the type 1 pattern, we propose that the compensatory flow has a rapid-onset coincident with the restriction of blood flow on the ipsilateral side, and this has the effect of both slowing and limiting the purine rise during carotid clamping.

Patients displaying the type 2 pattern may retain some ability for contralateral compensation; however, we suggest that the onset of the compensation is delayed—hence the tendency to higher initial increases in blood purines, and the later fall of purine levels during the clamp phase.

During the type 3 pattern, patients rapidly lost consciousness during carotid clamping. We suggest that they may have lost the ability to compensate with enhanced blood flow from the contralateral side. Furthermore, as their blood purines were high even at the pre-operative stage, their brains may be under chronic ischaemic stress. As the blood purine levels fell to below baseline levels within minutes of shunt insertion, the shunting may restore cerebral oxygen delivery either to, or even above, pre-clamp levels.

There is abundant evidence connecting brain ischaemia with the release of purines [31]. For example, in isolated brain slices, glucose-oxygen deprivation (an in vitro model of ischaemia) stimulates release of adenosine and downstream purines [7, 9, 11, 14]. In animal models, middle cerebral artery occlusion also causes adenosine release [8, 32, 33]. In humans, two studies suggest that adenosine levels in blood are elevated during CEA [21], and following stroke and TIA [20]. Nevertheless, the utility of purines as a measurement of brain ischaemia has largely been overlooked. One complication with the standard techniques to measure blood purines is that purine half-life in blood is only a few minutes. Accurate measurement of blood purines is thus difficult, as it requires rapid separation of cells and plasma, and inhibition of any reuptake mechanisms or degradative processes. It is perhaps this latter point that has made exploiting purines in clinical diagnosis difficult to date.

We believe that our methods overcome this drawback and have the potential to provide a point-of-care device for rapid measurement of purines in freshly drawn unprocessed blood. This may unlock the potential for purine measurements to be used to assess neurological status in stroke patients. How might this methodology change clinical practice? We suggest that purine measurements could be used to prioritize patients for CEA: patients with higher resting purine levels might benefit from earlier surgery. It would also appear that such patients might be more likely to require intraoperative shunting and, if required, shunt patency could be confirmed via the effect on arterial purine levels. Although we have not studied stroke patients, there are obvious potential applications here too. For example, a point-of-care device for measuring purines in the hands of first responders would enable the rapid identification of stroke, and complement prehospital tests for stroke, such as the Face Arms Speech Test, and CT scans, to give more accurate diagnosis. The faster and more accurate identification of stroke patients should in turn lead to more rapid treatment with better outcomes. In addition, as the purines are only produced during ongoing ischaemia, repeated measurements may allow assessment in real time as to whether the ischaemic injury is clearing or worsening without necessitating further brain scans.

We note that ischaemia, anywhere in the body, has the potential to cause elevation of purines in blood. Cardiac ischaemia is of particular interest as purine efflux during this condition is well documented [34, 35]. Purine efflux from the brain can also occur during traumatic brain injury [36] and seizures [31, 37, 38]. Optimal use of purines as a diagnostic aid will therefore always depend on other clinical measures and the overall clinical context of the patient.
